# The Impact of Imitation on Vaccination Behavior in Social Contact Networks

**DOI:** 10.1371/journal.pcbi.1002469

**Published:** 2012-04-12

**Authors:** Martial L. Ndeffo Mbah, Jingzhou Liu, Chris T. Bauch, Yonas I. Tekel, Jan Medlock, Lauren Ancel Meyers, Alison P. Galvani

**Affiliations:** 1 Department of Epidemiology and Public Health, Yale University School of Medicine, New Haven, Connecticut, United States of America; 2 Department of Mathematics and Statistics, University of Guelph, Guelph, Ontario, Canada; 3 Department of Ecology and Evolutionary Biology, Yale University, New Haven, Connecticut, United States of America; 4 Department of Mathematical Sciences, Clemson University, Clemson, South Carolina, United States of America; 5 Department of Biomedical Sciences, Oregon State University, Corvallis, Oregon, United States of America; 6 Section of Integrative Biology, The University of Texas at Austin, Austin, Texas, United States of America; 7 Santa Fe Institute, Santa Fe, New Mexico, United States of America; University of Michigan and Howard Hughes Medical Institute, United States of America

## Abstract

Previous game-theoretic studies of vaccination behavior typically have often assumed that populations are homogeneously mixed and that individuals are fully rational. In reality, there is heterogeneity in the number of contacts per individual, and individuals tend to imitate others who appear to have adopted successful strategies. Here, we use network-based mathematical models to study the effects of both imitation behavior and contact heterogeneity on vaccination coverage and disease dynamics. We integrate contact network epidemiological models with a framework for decision-making, within which individuals make their decisions either based purely on payoff maximization or by imitating the vaccination behavior of a social contact. Simulations suggest that when the cost of vaccination is high imitation behavior may decrease vaccination coverage. However, when the cost of vaccination is small relative to that of infection, imitation behavior increases vaccination coverage, but, surprisingly, also increases the magnitude of epidemics through the clustering of non-vaccinators within the network. Thus, imitation behavior may impede the eradication of infectious diseases. Calculations that ignore behavioral clustering caused by imitation may significantly underestimate the levels of vaccination coverage required to attain herd immunity.

## Author Summary

Both infectious diseases and behavioral traits can spread via social contacts. Using network-based mathematical models, our study addresses the interplay between these two processes, as disease spreads through a population and individuals copy their social contacts when making vaccination decisions. Imitation can produce clusters of non-vaccinating, susceptible individuals that facilitate relatively large outbreaks of infectious diseases despite high overall vaccination coverage. This may explain, for example, recent measles outbreaks observed in many countries with universal measles vaccination policies. Given that vaccine decisions are likely to be influenced by social contacts and that such imitation can have detrimental epidemiological effects, it is important that policy makers understand its causes, magnitude and implications for disease eradication.

## Introduction

Vaccination is the primary public health measure for preventing transmission of infectious diseases as well as reducing morbidity and mortality from infections [1]. An individual's decision-making with respect to vaccination may depend on perceived risk of infection, cost of infection, cost of vaccination, and the vaccinating behaviors of other individuals [2], [3], [4]. Game theory has been integrated into epidemiological models to investigate vaccination behaviors [5], [6], [7], [8]. Previous game-theoretic studies on vaccination dynamics typically assume that the population is homogeneously mixed and fully rational, defined as making decisions that yield the highest personal utility based on their perceived risks. In reality, there is individual heterogeneity in the number of contacts [9]–[22] and individuals frequently imitate behaviors of their contacts [23], [24], particularly those who appear to have adopted successful strategies [25], [26]. In addition, peer influence is a significant determinant of vaccine uptake in many populations [27].

An imitation vaccination model was previously developed under the assumption of a homogeneously mixed population [25], [28]. This model predicts that imitation is likely to generate oscillations in vaccine uptake, and that the oscillations tend to be large when the perceived risk of vaccination is high [25]. Since this model assumes that the population is homogeneously mixed, it cannot capture clustering of vaccination behaviors in a social network. The clustering of vaccination opinions can exacerbate disease outbreaks by interfering with herd immunity [29], [30], [31].

To evaluate the effect of imitation dynamics on vaccination and disease outbreaks, we develop social network models with imitation behavior. We consider three different contact network structures, a contact network based on a prior study of contact patterns within Vancouver [9], a relative homogeneous network with a Poisson degree distribution, and a heterogeneous scale free network (with a power law degree distribution). We assume that a portion of the population adopts vaccination based on a “payoff maximization” strategy that maximizes their perceived payoff, and the remaining population imitates the vaccination choices of their neighbors.

For all three networks considered, we find that imitation behavior increases the equilibrium level of vaccination coverage when vaccines are inexpensive and decreases vaccination coverage when vaccines are expensive. However, when imitation increases vaccination coverage, it simultaneously leads to connected clusters of unvaccinated individuals, which increase disease prevalence. The emergence of susceptible clusters and its detrimental epidemiological effects are most prominent when vaccination coverage is close to the herd immunity threshold.

## Methods

### Basic description

We consider a social contact network where individuals can switch between decisions of vaccinating or not vaccinating. An individual's vaccination decision is a function of both the strategies their neighbors have adopted and the perceived benefits of vaccination. Individuals know only the vaccination opinions of immediate neighbors (i.e., whether they are in favor of or opposed to vaccinating), and update their strategies either by imitating one of their neighbors (i.e., following their opinion) or by maximizing their perceived benefits. The fraction of individuals with imitation behavior is indicated by 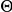, and the remaining individuals (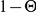) follow a payoff maximization strategy. The population opinion configuration is denoted by 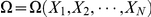, where 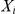 indicates the vaccination opinion of individual *i*,
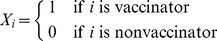
(1)Let 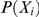 be the perceived payoff of an individual *i* with opinion 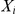, then
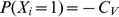
(2)

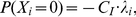
(3)where *C_V_* is the individual's cost of vaccinating, *C_I_* is the individual's cost of infection, and 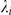 is perceived probability of infection. The payoffs are negative because maximizing a payoff in this context means minimizing a negative health cost/impact. Let 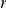 be the relative cost of vaccination (cost of vaccination/cost of infection), 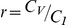. Without loss of generality, we can rewrite Eqs. 2 and 3 as:
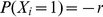
(4)

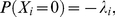
(5)Let 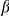 be the perceived probability of contracting the disease from an infectious neighbor at a given time step and 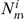 be the number of non-vaccinators in the neighborhood of *i*, respectively. We assumed that the perceived probability of infection depends on the number of non-vaccinator neighbors (whose status as non-vaccinators is assumed known), with no correlation between degree and the number of non-vaccinator neighbors. From basic probability theory we can express 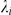 as
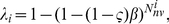
(6)where 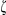 denotes the perceived probability that an unvaccinated neighbor will not become infected.

### “Payoff maximization” behavior

A payoff maximizer *i* will vaccinate if 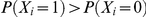 and will not vaccinate if 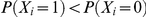. When 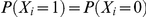, an individual *i* will adopt the vaccinator or non-vaccinator strategy with equal probability (50%). If the entire population adopts payoff maximization strategy, the system is expected to settle on the Nash equilibrium at steady state.

### Imitation vaccination behavior

**Figure 1:**
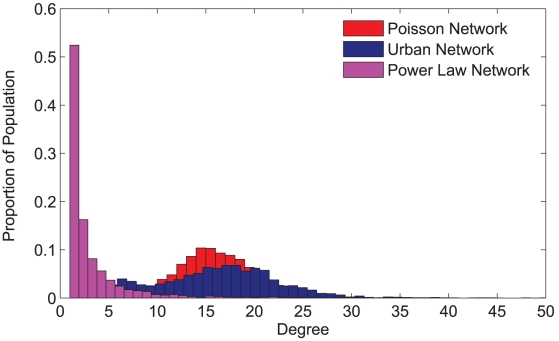
Degree distributions. The proportion of the population with each given degree are different for a Poisson network (red histogram), urban network (blue histogram), and exponentially-scaled power law network (pink histogram).

An imitator *i* randomly chooses a neighbor (‘role model’) *j* to imitate. Imitator *i* adopts *j*'s vaccination decision according to predetermined rules:

1. If *i* and *j* have the same opinion, they will both hold that opinion.
2. If *i* and *j* have opposite opinions, *i* will adopt *j*'s vaccination decision with probability
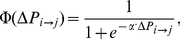
(7)where 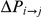 is the benefit that *i* gains by adopting *j*'s vaccination decision. If *i* is a vaccinator and *j* is a non-vaccinator, 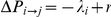; if *i* is a non-vaccinator and *j* is a vaccinator, 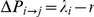. If we assume 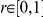, 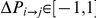. The parameter 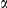 in Eq. 7 determines the individuals' responsiveness to payoff differences. For low values of 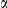, Eq. 7 changes more gradually as 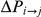 goes from negative to positive, meaning that imitators are less responsive to payoff differences, and an individual with high payoff may adopt the vaccination decision of a less successful role model. However, for high values of 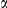, Eq. 7 changes abruptly at 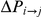 = 0, meaning that imitators are highly responsive to payoff differences (Figure 1). Eq. 7, also known as the Fermi function [26], [31], has been widely used to model strategy changes induced by imitation behavior [26], [31], [32].

### Contact networks

**Figure 2:**
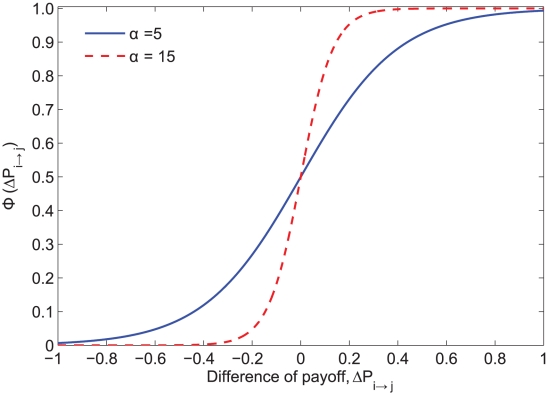
Fermi function. Probability that individual *i* adopts individual *j*'s vaccination strategy. α represents the degree to which individuals respond to the differences of payoff.

We compare vaccination dynamics across three different classes of networks: a pseudo-empirical urban network based on contact patterns within Vancouver, Canada [9], a homogeneous random network with a Poisson degree distribution, parameterized so that the average degree is equal to that of the urban network, and a highly heterogeneous, power law network in which degrees follow a truncated power law distribution. Let 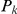 denote the probability that a randomly selected individual in a network has degree *k*. The Poisson network is given by 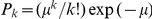
with mean contact number 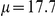; the power law network is given by 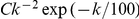 with mean contact number of 4.5 (Figure 2). We calibrate epidemic parameters to ensure that infection risk in an unvaccinated population is equal across all network structures [33], [34]. More precisely, we calibrate the value of disease transmission probability to ensure that the average final epidemic size is equal across the population structures. We chose the final size to be equal to 90%, although results were found to be qualitatively robust for a range of final sizes. For each network, the population size *N* was equal to 5000. The contact networks are generated using the configuration model (CM) algorithm for constructing finite random networks with a specified degree sequence [35], [36]. We generated degree sequences by choosing random deviates from these degree distributions.

To investigate the effect of imitation behavior on vaccination and disease outbreaks, we assumed that the perceived transmission probability 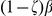 is equal to the transmission probability of the infectious disease.

### Monte Carlo simulations

We perform Monte Carlo simulations on vaccinating opinion formation and disease transmission according to these four steps:

- Generate the contact network
- Randomly sample 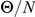 individuals from the entire population *N* and assume they are imitators. Imitators will update their vaccination decisions according to the imitation model. The rest of the population updates their decisions so as to maximize their perceived benefits of vaccination. We vary 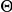 from 0 to 1.
- Randomly assign each individual a vaccination decision (i.e., vaccinate or not vaccinate) such that the initial vaccination coverage is between 0% and 100%, and then run the model, using a parallel update rule, until a steady state is reached. The steady state is reached when the difference in mean vaccination coverage over three consecutive time windows (*N* steps) is sufficiently small (<0.005). Each individual gets vaccinated according to its vaccination strategy. Vaccinated individuals are immune to infection.
- Run the standard SIR (susceptible – infected – removed) epidemic model on the network generated in step (i) with the final vaccination decisions reached in step (iii). The infection is introduced by inoculating 10 randomly chosen susceptible individuals to minimize stochastic fadeout. At each time step, each susceptible individual *i* is infected with probability 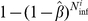, where 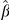 is the transmission probability of the infectious disease and 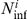 is the number of infected neighbors of individual *i*. An infected individual recovers and becomes immune with probability 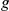 per time step. We run the model until no infection exists in the system.
- In our analyses, baseline parameter values (Table 1) are used unless stated otherwise. We vary the relative cost of vaccination from 0 to 1. The equilibrium results represent the averages over 100 iterations of step (iv) in 100 independent simulations (step (ii)–(iv)).

**Table 1:** Summary of the parameters used in simulations.

Parameter	Description	Baseline value
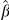	Transmission probability	Calibrated*
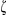	Probability a random contact is not infected per epidemic	Calibrated**
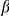	Perceived transmission probability	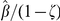
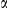	Degree of responsiveness to differences of payoff	5
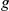	Recovery probability	0.25
*N*	population size (number of nodes)	5000
*r*	Relative cost of vaccination	variable of interest
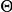	Fraction of imitators	variable of interest

*For each network structure, transmission probability was chosen so as to ensure that the average final size of the epidemic is approximately equal to 90% of the total population. For Poisson network 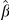 = 0.05, urban network 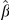 = 0.06, and exponential-scaled power law network 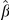 = 0.52.

**The perceived probability a random contact is infected per epidemic 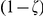 was chosen to be constant, with value varying between 50–90%. For each value of 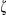, 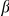 was chosen such that 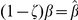.

## Results

### Equilibrium vaccination coverage

**Figure 3:**
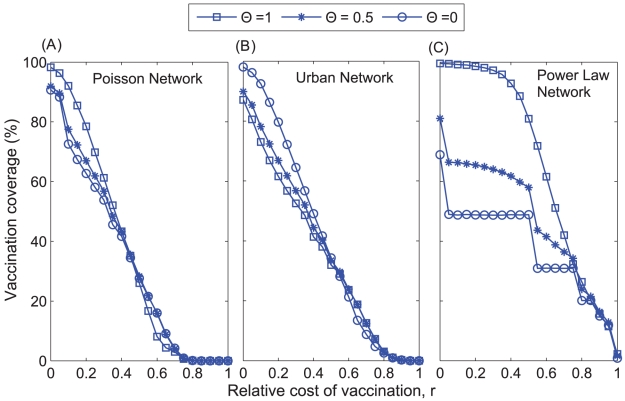
Vaccination coverage as a function of the relative cost of vaccination (*r*) and the fraction of imitators (Θ) in a (A) homogenous Poisson network, (B) urban network, and (C) exponentially-scaled power law network. Parameters: Population size *N* = 5000, recovery rate *g* = 0.25 d^−1^, α = 5.

We found that imitation (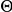) tends to increase vaccination coverage when the cost of vaccination (*r*) is low and to decrease vaccination coverage when *r* is high (Figure 3). However, the effect of imitation varies with the degree of distribution of the contact network (Figure 3). Comparing two extreme cases of 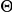 (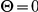: fully payoff maximization and 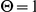: fully imitation), imitation dynamics (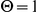) can promote near-universal coverage when the cost of vaccination is very low compared to that of infection (small values of *r*) (Figure 3). This difference is particularly pronounced in the power law network.

**Figure 4:**
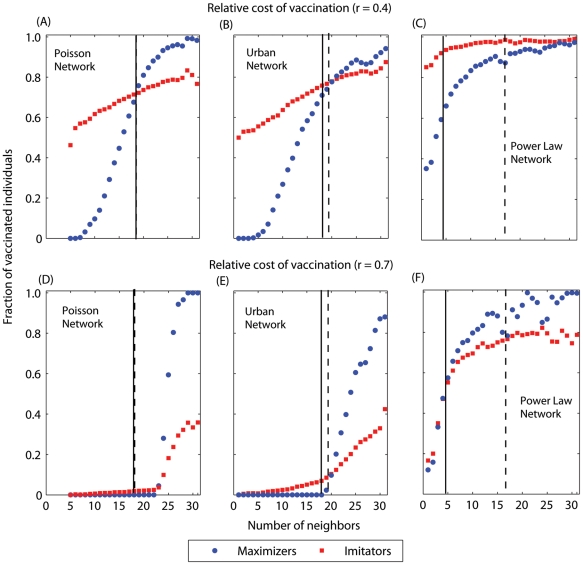
Frequency of vaccination as a function of the number of neighbors an individual has for the two extreme cases: fully payoff maximization (Θ = 0) and fully imitation (Θ = 1). The homogeneous Poisson network is represented by (A,D), the urban network by (B,E), and the exponential-scaled power law network by (C,F). (A–C) the relative cost of vaccination *r* = 0.1; (D–E) *r* = 0.7. The solid line represents the average degree of the network, and the dashed line represents the average excess degree of the network. The average excess degree is a measure of the tendency to which individuals with high degree are connected to individuals with low degree, and *vice versa* [11].

Individuals have a high incentive to vaccinate when the relative cost of vaccination (*r*) is low, and not to vaccinate when it is high. Moreover, when most of an individual's neighbors adopt a given strategy, an individual has more incentive to adopt the opposite strategy. That is, if an individual is surrounded by vaccinators, their risk of infection and resulting incentive to vaccinate will both be low; if an individual is surrounded by non-vaccinators, their risk of infection and incentive to vaccinate will be high. However, imitators have a non-zero probability of copying the vaccination strategy that is adopted by most of their neighbors, even when such a strategy may be less suboptimal for them. For low values of *r*, payoff maximizers have a high incentive to vaccinate, and thus imitators are likely to have vaccinators as role models; the opposite should be true under high values of *r*. Therefore, for low values of *r*, imitators may have higher vaccination coverage than payoff maximizers, whereas for high values of *r*, imitation may lead to fewer vaccinators than anticipated by payoff maximization strategy (Figures 3 and 4).

**Figure 5:**
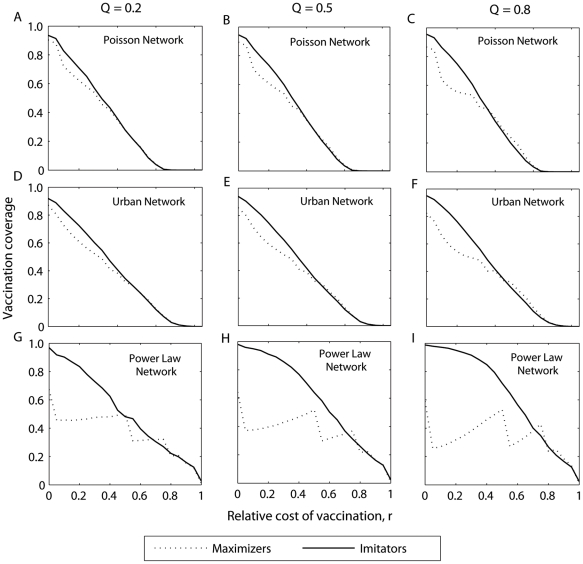
Vaccination coverage of imitators and payoff maximizers as a function of the relative cost of vaccination (*r*) for the portion of imitators equals to Θ = 0.2, Θ = 0.5, Θ = 0.8. The homogeneous Poisson network is represented by (A,B,C), the urban network by (D,E,F), and the exponential-scaled power law network by (G,H,I).

The power law network was shown to be more sensitive to the effect of imitation behavior than Poisson and Urban networks (Figures 3 and 4). This is due to the fact that the power law network has a highly skewed degree distribution, with a small density of highly-connected individuals. Highly-connected individuals (hubs) have a high incentive to vaccinate, whereas individuals with few contacts have less incentive to vaccinate. By imitating their highly-connected neighbors, individuals with few contacts become more likely to vaccinate, which may substantially increase vaccination coverage (Figures 3 and 5). However, this increase of vaccination coverage overall decreases the incentive for hubs to vaccinate (Figure 5). Depending on the density of hubs and the value of the relative cost of vaccination, this decrease in the incentive of hubs to vaccinate may reduce the total vaccination coverage within the population (Figure 3).

### Final size of outbreak

**Figure 6:**
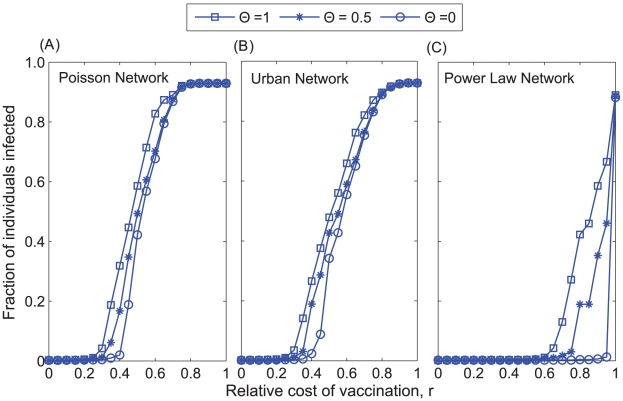
Epidemic size as a function of the relative cost of vaccination (*r*) and the fraction of imitators (Θ) in a (A) homogenous Poisson network, (B) urban network, and (C) exponentially-scaled power law network.

**Figure 7:**
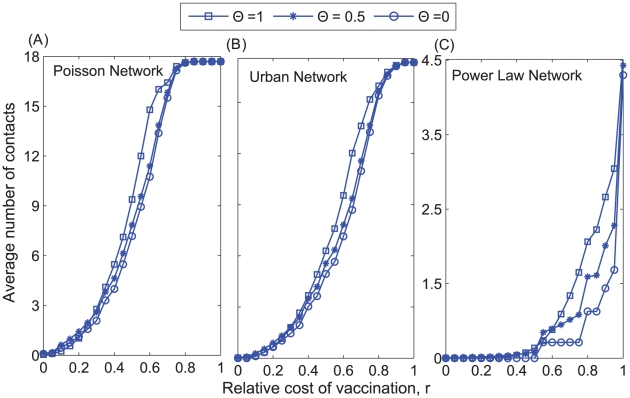
Average number of contacts between non-vaccinators as a function of the fraction of imitators (Θ) and the relative cost of vaccination (*r*) in a (A) homogenous Poisson network, (B) urban network, and (C) exponentially-scaled power law network.

We found that imitation (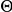) increases the final size of the outbreak (i.e., the fraction of the population infected) for intermediate costs of vaccination (*r*) (Figure 6). Numerical investigation showed that this range of values, which varies with the contact network (Figure 6), has an upper bound that represents the value of *r* above which it is disadvantageous for anyone to vaccinate, resulting in a full blown epidemic, and a lower bound which represents the value of *r* below which the average final epidemic size was less than twice the size of the initial inoculum of 10 infected individuals. Imitation dynamics can increase the vaccination coverage relative to a population with payoff maximization strategy, when the cost of vaccination is low, but can never decrease the final epidemic size (Figures 3 and 6). As a result of behavioral clustering that emerges from imitation dynamics, the size of the epidemic does not necessarily decrease as vaccination coverage increases. That is, vaccinators tend to contact vaccinators, and non-vaccinators tend to contact non-vaccinators (Figure 7). Because herd immunity is considerably high in these pockets of vaccinators, further vaccination within these pockets reduces transmission to a lesser degree than if vaccination were increased in regions of the network with relatively low vaccination coverage. The clusters of non-vaccinators fuel transmission and increase the probability of an outbreak. This effect of imitation is most prominent when vaccination coverage is close to the herd immunity threshold (Figures 3 and 6).

### Sensitivity analysis for the responsiveness to differences of payoff

**Figure 8:**
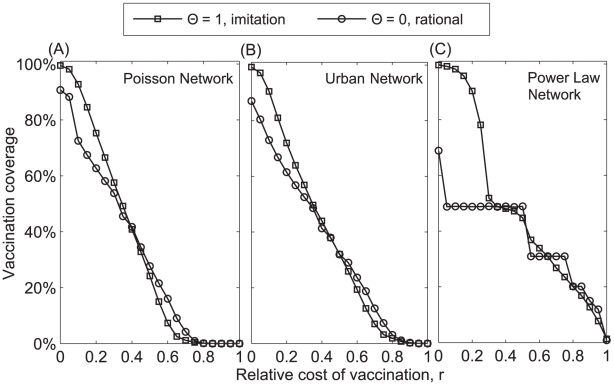
Vaccination coverage under strong responsiveness to differences of payoff (α = 15) in a (A) homogenous Poisson network, (B) urban network, and (C) exponentially-scaled power law network. Vaccine coverage is given as a function of *r* for the two extreme cases: fully payoff maximization (Θ = 0) and fully imitation (Θ = 1). Parameters are identical to Table 1, except α = 15.

To investigate the sensitivity of our results to the degree to which imitators respond to payoff differences between themselves and their neighbors, we compared weak responsiveness to strong responsiveness (Figure 2). For strong responsiveness, individuals reliably copy the strategy of successful neighbors. However, if most neighbors of an imitator adopt a given strategy, then the opposite strategy becomes advantageous, and the imitator would be more likely to choose the opposite strategy. Strong responsiveness, relative to weak responsiveness, leads imitators to rarely copy unsuccessful neighbors (Figure 2). Therefore, as the degree of responsiveness increases (*α* = 15), vaccination coverage under pure imitation (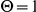) tends to converge towards the vaccination level predicted by the payoff maximization equilibrium (Figures 3 and 8). A similar convergence occurs for final epidemic size (result not shown here).

## Discussion

Classic economic theory has not considered the reality that individuals frequently imitate others [2], [3], [24], [31], [37]. Imitation begins with simple behaviors in infancy and evolves into more complex behaviors in childhood and adulthood [31], [37]. In the context of epidemiology, imitation behavior can influence vaccination patterns and thus the dynamics of disease outbreaks [2], [3], [24]. In this work, we address the impact of imitation on vaccination coverage, disease prevalence, and the herd immunity threshold. We develop a model that allows contact patterns to be heterogeneous and individuals to incorporate varying degrees of imitation into decision-making. Individuals within a social contact network can switch between the strategies of vaccinating and not vaccinating. An individual's decision regarding whether to vaccinate is affected by the strategies that their neighbors have adopted or the perceived net benefits of vaccination. Monte Carlo simulations show that imitation dynamics increase the equilibrium vaccination coverage when vaccination cost is relatively low and may decrease vaccination coverage when vaccination is costly. In both cases, imitation actually exacerbates disease transmission when vaccination is inexpensive through the social clustering of non-vaccinators. The detrimental effects of imitation are most prominent when the vaccination is close to the herd immunity threshold.

Salathe and Bonhoeffer recently developed a vaccination opinion formation model to reveal that opinion clustering increases the size of an epidemic [29]. Their model assumed that opinions are determined by the proportion of neighbors that have the same opinion about vaccination, such that whenever an individual switches opinion, another individual has to switch the opinion in an opposite way in order to maintain constant vaccination coverage level [29]. Extending this previous seminal model, we consider both imitation (opinion formation) and payoff maximization consideration (individuals are not just blindly imitating neighbors; they are trying to optimize a payoff function). Our model thereby recognizes that vaccine decision-making is not a purely imitative process, and often depends on actual health considerations. Additionally, by incorporating payoffs, we are able to analyze the impact of vaccine cost on the dynamics of vaccination.

This analysis takes an initial step towards understanding the combined impacts of payoff maximization and imitative decision-making on vaccination coverage and epidemiological dynamics. The model, however, rests on several simplifying assumptions. For example, the contact networks are assumed to remain static throughout the epidemic, and to be identical for both disease and behavioral transmission. These assumptions could be relaxed by incorporating temporal changes in network structure [38], and modeling multiple different edge types (e.g. individual variation in susceptibility and infectivity) [39]. The model can also be extended to allow individuals to follow mixed vaccination strategies, or by incorporating the effects of past epidemics on vaccine decision-making [40].

We find that imitation leads to clustering of susceptible individuals, which may exacerbate outbreaks of infectious diseases. For example, imitation may explain how outbreaks of measles have occurred in countries with high overall vaccination coverage [26], [29], [41], [42]. Given that vaccine decisions are likely to be influenced by social contacts [29], [32], [41] and that such imitation can have detrimental epidemiological effects [29], it is important that policy makers understand its causes, magnitude, and implications for disease elimination.

Our findings indicate that the common assumptions of simple payoff maximization and homogeneous mixing can lead to misestimates of the level of vaccination coverage necessary to control a disease outbreak. Our model provides a general framework for investigating the effect of imitation on vaccination decision-making and disease outbreaks. The model can be applied to study the interactions between behavior, public health, and epidemic dynamics for specific infectious diseases. Data describing real world imitation behavior in vaccination decision-making will be critical to future public health applications of the model.
